# Blood Phenylalanine Control in Paediatric and Adult Centres in the UK: Data from 2012–2018

**DOI:** 10.3390/nu18132069

**Published:** 2026-06-24

**Authors:** Alex Pinto, Catherine Ashmore, Jane Ash, Barbara Cochrane, Duncan Cole, Sarah Bailey, Clare Dale, Anne Daly, Charlotte Dawson, Sharon Evans, Sarah Firman, Suzanne Ford, Anne Grimsley, Diane Green, Tarekegn Geberhiwot, Sarah Howe, Inderdip Hunjan, Fatma Ilgaz, Richard Jackson, Nicola McStravick, Camille Newby, Natalia Oxley, Radha Ramachandran, Katie Rawlins, Louise Robertson, Danja Schulenburg-Brand, Kalpana Shah, Hugh Lemonde, Rachel Skeath, Allyson Terry, Gisela Wilcox, Alison Woodall, Karen Van Wyk, Júlio César Rocha, Anita MacDonald

**Affiliations:** 1 Birmingham Children’s Hospital, Steelhouse Lane, Birmingham B4 6NH, UK; catherine.ashmore@nhs.net (C.A.); a.daly3@nhs.net (A.D.); sharon.morris6@nhs.net (S.E.); anita.macdonald@nhs.net (A.M.); 2 All Wales Inherited Metabolic Disease Service, University Hospital of Wales, Heath Park Way, Cardiff CF14 4XW, UKduncan.cole@wales.nhs.uk (D.C.); sarah.bailey3@wales.nhs.uk (S.B.); danja.schulenburg-brand@wales.nhs.uk (D.S.-B.); 3 NHS Greater Glasgow and Clyde, Royal Hospital for Children, Govan Rd, Glasgow G51 4TF, UK; 4 University Hospital Birmingham, Mindelsohn Way, Birmingham B15 2GW, UK; clare.dale@uhb.nhs.uk (C.D.); charlotte.dawson@uhb.nhs.uk (C.D.); tarekegn.geberhiwot@uhb.nhs.uk (T.G.); sarah.howe@uhb.nhs.uk (S.H.); louise.robertson@uhb.nhs.uk (L.R.); 5 Guy’s & St Thomas’ NHS Foundation Trust, Westminster Bridge Rd, London SE1 7EH, UK; sarah.firman@gstt.nhs.uk (S.F.); radharamachandran@nhs.net (R.R.);; 6 North Bristol Trust, Southmead Rd, Bristol BS10 5NB, UK; suzanne.ford@nbt.nhs.uk; 7 Royal Belfast Hospital for Sick Children, 274 Grosvenor Rd, Belfast BT12 6BA, UK; anne.grimsley@belfasttrust.hscni.net; 8 Salford Royal NHS Foundation Trust, Stott Ln, Salford M6 8HD, UKgisela.wilcox@manchester.ac.uk (G.W.); alison.woodall@nca.nhs.uk (A.W.); 9 St Lukes Hospital, Little Horton Ln, Bradford BD5 0NA, UK; inderdip.hunjan@bthft.nhs.uk (I.H.); natalia.oxley@bthft.nhs.uk (N.O.); 10 Department of Nutrition and Dietetics, Faculty of Health Sciences, Hacettepe University, 06100 Ankara, Turkey; fatmacelik86@gmail.com; 11 Department of Health Data Science, University of Liverpool, Liverpool L69 3GJ, UK; richj23@liverpool.ac.uk; 12 Belfast Health and Social Care Trust, The Royal Hospitals Belfast, Grosvenor Road, Belfast BT12 6BA, UK; nicola.mcstravick@belfasttrust.hscni.net; 13 Bristol Royal Hospital for Children, Upper Maudlin St, Bristol BS2 8BJ, UK; camille.newby@uhbw.nhs.uk; 14 Evelina Children’s Hospital, Westminster Bridge Rd, London SE1 7EH, UK; kalpana.shah2@nhs.net (K.S.); hugh.lemonde@nhs.net (H.L.); 15 Great Ormond Street Hospital, Guilford St, London WC1N 3BH, UK; rachel.skeath@gosh.nhs.uk; 16 Alder Hey Children’s Hospital, E Prescot Rd, Liverpool L14 5AB, UK; allyson.terry1@nhs.net; 17 School of Medical Sciences, Faculty of Biology Medicine & Health, University of Manchester, Oxford Road, Manchester M13 9PL, UK; 18 Royal Manchester Children’s Hospital, Oxford Rd, Manchester M13 9WL, UK; karen.vanwyk@mft.nhs.uk; 19 Nutrition and Metabolism, NOVA Medical School (NMS), Faculdade de Ciências Médicas (FCM), Universidade Nova de Lisboa, 1169-056 Lisboa, Portugal; rochajc@nms.unl.pt; 20 Centro de Investigação em Tecnologias e Serviços de Saúde (CINTESIS), NOVA Medical School (NMS), Faculdade de Ciências Médicas (FCM), Universidade Nova de Lisboa, 1169-056 Lisboa, Portugal; 21 Reference Centre of Inherited Metabolic Diseases, Unidade Local de Saúde São José, 1169-045 Lisboa, Portugal; 22 Comprehensive Health Research Centre (CHRC), NOVA Medical School (NMS), Faculdade de Ciências Médicas (FCM), Universidade Nova de Lisboa, 1169-056 Lisboa, Portugal

**Keywords:** phenylketonuria, phenylalanine, protein, European guidelines

## Abstract

**Background:** Metabolic control in phenylketonuria (PKU) is known to deteriorate with age, but national-level data describing blood phenylalanine (Phe) control across the United Kingdom (UK) are limited. **Objective:** To characterise blood Phe control in individuals with PKU attending UK metabolic centres. **Methods:** Sixteen UK centres (nine paediatric, six adult, one mixed) retrospectively extracted blood Phe results collected between 2012 and 2018. Demographic, phenotypic and monitoring-related variables were analysed. Written consent for data collection was obtained from all patients or their caregivers. **Results:** Data were available for 871 individuals (55% female), of whom 744 (85%) were classified as follows: classical PKU, 75%, mild PKU, 22% and hyperphenylalaninaemia, 3%. Mean blood Phe concentrations were significantly higher in adults than children (491 ± 308 vs. 303 ± 199 µmol/L; *p* < 0.001), and the proportion of samples within target range declined steadily with age, from 78% in children < 2 years to 36% in adults ≥ 41 years. Individuals with classical PKU had higher mean Phe concentrations and lower target attainment than those with HPA (386 vs. 300 µmol/L; 61% vs. 78%; *p* < 0.001), while mild PKU and HPA showed comparable control. Females generally demonstrated better metabolic control than males. More frequent dried blood spot sampling for blood Phe was strongly associated with improved metabolic control: weekly (254 ± 175 µmol/L; 82% within target), fortnightly (319 ± 207 µmol/L; 70%), monthly (397 ± 231 µmol/L; 61%), and less than monthly (624 ± 349 µmol/L; 44%). Nearly half of the blood Phe samples (47%) with recorded timing were taken in a non-fasting state. **Conclusions:** Achieving lifelong metabolic stability on a Phe-restricted diet alone remains challenging. These national data highlight the need for broader therapeutic options to support individuals with PKU across the lifespan.

## 1. Introduction

Phenylketonuria (PKU; McKusick #261600) is an inherited disorder of phenylalanine (Phe) metabolism caused by pathogenic variants in the phenylalanine hydroxylase (PAH) gene [1,2,3,4]. Reduced PAH activity impairs the conversion of Phe to tyrosine (Tyr), leading to elevated blood Phe concentrations [3]. Most PAH variants are missense mutations that impair enzyme function through protein misfolding or reduced catalytic efficiency, although nonsense, frameshift, and splice site mutations also occur [5,6]. The type and location of the underlying mutation influence residual PAH activity and determine clinical severity, contributing to the broad phenotypic spectrum observed in PKU [6].

In the absence of genotype data, PKU severity is commonly classified according to untreated blood phenylalanine (Phe) concentrations: 120–600 µmol/L for hyperphenylalaninaemia (HPA), 600–1200 µmol/L for mild PKU (mPKU), and >1200 µmol/L for classical PKU (cPKU) [2]. Alternative classification frameworks have been proposed, including the most recent revision of the European PKU guidelines, which incorporates responsiveness to a pharmacological co-factor. Under this system, PAH deficiency is categorised as: not requiring treatment (Phe < 360 µmol/L), requiring treatment and co-factor responsive, or requiring treatment and co-factor unresponsive [7].

Blood phenylalanine (Phe) concentration is the primary biochemical marker used to assess metabolic control in PKU [7,8]. In the absence of treatment, Phe accumulates in the blood and enters the brain by competing with large neutral amino acids (LNAAs) for carrier-mediated transport across the blood–brain barrier [9,10,11]. This reduces cerebral LNAA availability and impairs neurotransmitter synthesis from Tyr and tryptophan [12]. Elevated brain Phe is associated with reduced dopamine and serotonin levels [13,14,15], impaired cerebral protein synthesis, decreased cholesterol and myelin production, altered glutamatergic synaptic transmission, oxidative stress, disrupted glucose metabolism, and the formation of amyloid-like fibrils [16]. Sustained high Phe concentrations result in neurocognitive impairment, intellectual disability, fine motor deficits and seizures [17]. High Phe concentrations are also linked to difficulties in social and executive functioning, as well as behavioural and psychiatric symptoms, reflecting the multiple pathophysiological mechanisms underlying brain dysfunction in PKU [17].

Adverse outcomes can be mitigated when treatment is initiated promptly following identification through newborn screening [7]. The core management is a lifelong Phe-restricted diet, achieved by limiting natural protein intake and supplementing with a low-Phe protein substitute based on amino acids or glycomacropeptide [18,19,20]. A subset of individuals with residual PAH activity may benefit from pharmacological chaperone therapies (sapropterin or sepiapterin) [21,22,23], while pegvaliase is an additional option for individuals aged ≥12 years with blood Phe concentrations ≥ 600 µmol/L [24,25,26]. However, access to these pharmacological therapies varies substantially across countries.

Early evidence of age-related deterioration in metabolic control was reported by Walter et al. [27] in 2002, who examined blood Phe concentrations in children and adolescents with PKU in the UK and Australia. Using age-specific targets (<360 μmol/L for <5 years, <480 μmol/L for 5–10 years, and <700 μmol/L for >10 years), they observed a progressive decline in the proportion of results within the recommended range: 72% at ages 0–4 years and 73% at 5–9 years, falling to 50% at 10–14 years and 21% at 15–19 years [27]. More recently, Jurecki et al. [28] similarly demonstrated a consistent age-related decline in metabolic control when applying a uniform threshold of <360 μmol/L across all age groups.

Ahring et al. [29] reported similar findings across 10 European centres, although target ranges used to define optimal metabolic control varied between sites. More recently, metabolic control data from nine European centres (including one UK centre and one centre in Turkey) were analysed for the period 2012–2018 using the European PKU guideline thresholds (<360 μmol/L for individuals <12 years and <600 μmol/L for those ≥12 years). In this cohort, more than 73% of blood Phe measurements in infancy and childhood were within the recommended target. The proportion increased to 83% during adolescence, reflecting the higher upper limit of the target range for this age group [29]. In adulthood, however, metabolic control declined, with 64% of measurements within range at 19–30 years, 59% at 31–40 years, and only 40% in individuals >41 years [29].

In the UK, many PKU centres do not fully implement the blood Phe targets recommended in the European guidelines. Prior to the introduction of the 2017 and, subsequently, the 2025 European PKU guidelines, several UK centres operated with substantially higher therapeutic Phe ranges. In 1993, a working party of the UK Medical Research Council advised maintaining blood Phe concentrations between 120 and 360 µmol/L in young children, while permitting levels up to 400 µmol/L in school-aged children. For adolescents and adults, a target of ≤700 µmol/L was recommended. These higher thresholds reflected prevailing national practice and were shaped by the limited availability of adult metabolic services, challenges in sustaining strict dietary adherence across the lifespan, and the pragmatic clinical view at the time that higher Phe concentrations in adulthood were acceptable in the absence of robust long-term outcome data.

Routine psychological support, an essential component of multidisciplinary care, is not consistently available, and lifelong treatment is not universally endorsed by all healthcare professionals [30,31]. In a 2023 online survey developed by the NSPKU to assess the experiences of adults with PKU, Ilgaz et al. [30] reported that only 24% of respondents felt they received adequate support, highlighting substantial gaps in service provision.

In this multicentre, retrospective, longitudinal study we aimed to characterise metabolic control in individuals attending both paediatric and adult PKU centres across the UK.

## 2. Materials and Methods

### 2.1. Participating Centres

Metabolic dietitians from the British Inherited Metabolic Disease Group Dietitians Group (BIMDG-DG), representing all UK centres providing care for individuals with PKU, were invited to participate. Sixteen centres contributed data: nine paediatric centres, six adult centres, and one centre providing both paediatric and adult services. For analysis and presentation, each centre was assigned a unique identifier (A–P).

### 2.2. Patient Selection

**Figure 1:**
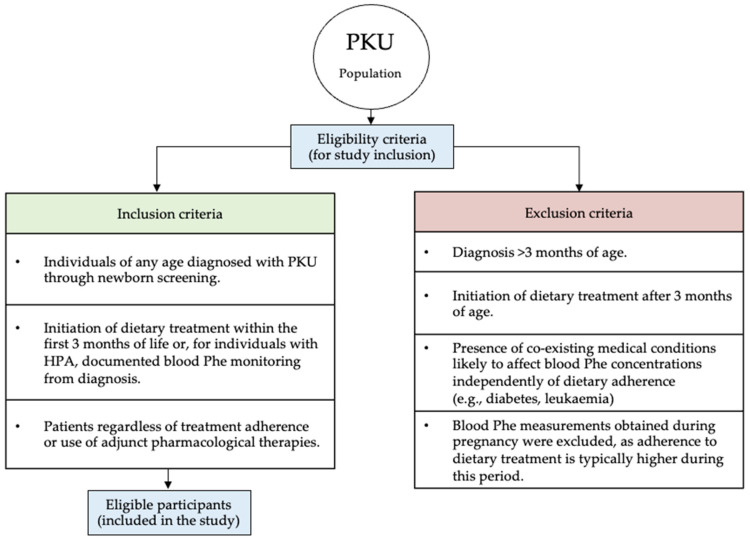
Flowchart of study inclusion and exclusion criteria. Abbreviations: PKU, phenylketonuria; HPA, hyperphenylalaninaemia.

Inclusion criteria were individuals of any age diagnosed with PKU through newborn screening; initiation of dietary treatment within the first 3 months of life or, for individuals with HPA, documented blood Phe monitoring from diagnosis; and patients regardless of treatment adherence or use of adjunct pharmacological therapies (Figure 1).

Exclusion criteria were: diagnosis >3 months of age; initiation of dietary treatment after 3 months of age; and the presence of co-existing medical conditions likely to affect blood Phe concentrations independently of dietary adherence (e.g., diabetes, leukaemia). Blood Phe measurements obtained during pregnancy were excluded, as adherence to dietary treatment is typically higher during this period.

### 2.3. Study Design

This multicentre, longitudinal, retrospective study included data collected between 2012 and 2018 comprising blood Phe measurements from individuals with PKU. The study period was selected to capture metabolic control data following the last European report describing control published in 2011 [29] and extending beyond the release of the first European PKU guidelines. Additional complementary variables were collected where available, including time of sampling, PAH variants, demographic characteristics, diagnostic blood Phe concentrations, dietary intake (prescribed natural protein, protein equivalent from protein substitutes, and total protein intake), use of adjunct therapies (e.g., sapropterin), anthropometric measures (weight and height), use of vitamin, mineral and energy supplements, and concomitant medications.

The present manuscript focuses exclusively on metabolic control outcomes from UK centres. Dietary data will be reported separately.

### 2.4. Procedures

All data were extracted from electronic or paper-based handwritten medical and dietetic records by A.P. across all participating centres. The objective of this manuscript was to analyse and describe overall metabolic control in individuals with PKU receiving care in specialised UK centres. Analyses were conducted according to PKU severity, sex, age group and frequency of blood Phe monitoring. Sapropterin was not available through the UK NHS (National Health Service) until late 2021, and only a small number of individuals were prescribed it during the study period, typically within research contexts. Consequently, no separate analyses based on sapropterin treatment were performed.

Optimal metabolic control was defined according to the European PKU guidelines: blood Phe concentrations between 120 and 360 μmol/L for individuals <12 years and between 120 and 600 μmol/L for those ≥12 years [7].

Classification of PKU severity was based on PAH variant analysis using the BIOPKU database [32]. When variant data were unavailable, diagnostic blood Phe concentrations were used to determine severity: hyperphenylalaninaemia (HPA) was defined as <600 μmol/L, mild PKU (mPKU) as 600–1200 μmol/L, and classical PKU (cPKU) as >1200 μmol/L [2].

The analysis followed the same methodology and was conducted in parallel with a European multicentre study that collected comparable data over the same period [33]. All blood Phe measurements from participating centres were combined into a single pooled dataset for national-level analysis. To achieve this, individual-level Phe results were collected in a standardised format from each centre by one research dietitian (A.P.).

### 2.5. Statistical Analysis

A formal sample size calculation was not undertaken, as the study aimed to recruit all patients meeting the predefined inclusion and exclusion criteria. Blood Phe concentration served as the primary outcome measure.

Categorical variables were summarised as frequencies and percentages, while continuous variables were reported as mean ± SD or median (range), as appropriate. Phe data were analysed using longitudinal regression techniques to account for repeated measures. Results were obtained in terms of the mean difference and 95% confidence intervals. Statistical significance was determined by a *p*-value of <0.05 throughout. Interpersonal variations were considered in overall mean results. All analyses were performed by R.J. using R software (version 3; R Foundation for Statistical Computing, Vienna, Austria).

### 2.6. Ethical Aspects

This study was conducted in accordance with the principles of the Declaration of Helsinki (as last amended by the 64th World Medical Association General Assembly, Fortaleza, Brazil, October 2013) and Good Clinical Practice guidelines. Ethical approval was granted by the West Midlands Black Country Research Ethics Committee (reference 18/WM/0188) and the Integrated Research Application System (IRAS; number 237853, approved 18 June 2018). Written informed consent, and age-appropriate assent where applicable, was obtained from all participants and/or their caregivers prior to data collection.

## 3. Results

### 3.1. Treatment Centres

Sixteen PKU centres across the UK participated in this study. Nine centres provided paediatric care only (centres A, C, D, E, G, I, J, K and N), six were adult centres (centres B, F, H, L, M and O), and one centre (centre P) provided both paediatric and adult services (Table 1).

Data were collected from 871 individuals with PKU, with centre-level sample sizes ranging from 8 to 129 participants. The majority of participants were recruited from paediatric centres, representing 59% of the total cohort (*n* = 513/871).

Overall, 61% of all eligible patients attending the participating centres were enrolled in the study, with centre-level participation rates ranging from 20% to 100%. All eligible individuals were invited to participate, and enrolment was contingent on the provision of consent and/or assent.

Blood Phe monitoring was conducted in all centres using dried blood spot (DBS) samples collected at home and posted to hospital laboratories for analysis. DBS Phe analysis is conducted by flow injection analysis–tandem mass spectrometry (FIA-MS/MS). This offers a rapid turnaround and high sample throughput.

The number of DBS returned from participants from each centre are given in Table 1.

### 3.2. Participant Age and Characteristics

Participants ranged in age from 1 to 70 years during the data collection period. A total of 871 individuals were included comprising 513 children (0–18 years) and 358 adults (>18 years) (Table 2). Among the children, 242 were female and 271 were male. Among the adults, 236 were female and 122 were male.

Diagnostic Phe levels and PAH variant data required to determine PKU severity were available for 85% of patients (744/871). Among these, the majority had classical PKU (cPKU; 75%, *n* = 560/744), 22% (*n* = 160/744) mild PKU (mPKU) and 3% (*n* = 24/744) had HPA. Not all individuals with HPA were receiving dietary treatment at the time of this study.

Most patients were managed with dietary treatment alone (857/871; 98%). Fourteen paediatric patients received sapropterin, either within research studies or on compassionate grounds.

### 3.3. Blood Phenylalanine Concentrations

Overall, 358 adults (29,387 blood spot samples) and 513 children (62,479 blood spot samples) were included in the analysis. Mean blood Phe concentrations were significantly lower in children (303 ± 199 μmol/L), with 71 ± 46% of measurements within therapeutic target range compared with adults (491 ± 308 μmol/L; 59 ± 49% within target; *p* < 0.001).

Mean blood Phe concentrations across paediatric centres (ages 0–18 years) ranged from 239 to 370 μmol/L, with standard deviations between 157 and 244 μmol/L. The proportion of blood Phe measurements within the therapeutic targets defined by the European PKU guidelines ranged from 56% to 83% across centres. In the adult centres, mean blood Phe concentrations ranged from 434 to 667 μmol/L, with standard deviations between 260 and 388 μmol/L. The proportion of results within target ranged from 47% to 71%. Across both paediatric and adult services, most centres reported <70% of blood Phe measurements within the recommended therapeutic range.

**Figure 2:**
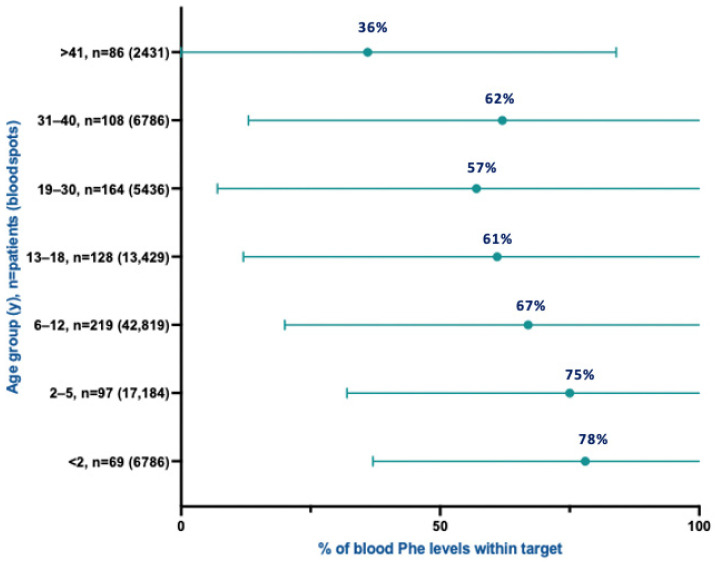
Mean percentage of blood Phe concentrations within target range for each age group.

Figure 2 illustrates the percentage of blood Phe measurements within the target range across age groups. The highest proportion of results within target was observed in children under 2 years of age (78%), with a progressive decline across older age groups, reaching a low of 36% among individuals aged >41 years.

Table 3 presents both the mean blood Phe concentrations and the mean percentage of measurements within the target range for each age group. Mean blood Phe levels increased progressively with age, rising from 272 ± 226 μmol/L in children under 2 years to a peak of 750 ± 362 μmol/L in individuals aged over 41 years. Variability in blood Phe concentrations also increased with age, with the standard deviation widening from 226 μmol/L in those under 2 years to 362 μmol/L in the oldest age group.

### 3.4. Blood Phenylalanine Concentrations by Sex

Table 4 presents mean blood Phe concentrations and the proportion of measurements within the therapeutic target range by age group, stratified by sex. No significant sex-related differences were observed in children aged <2 years (*p* = 0.387) or those aged 6–12 years (*p* = 0.212). In early childhood (2–5 years), males had statistically higher mean Phe levels than females (287 vs. 283 μmol/L; *p* < 0.001), whereas during adolescence females had significantly higher mean Phe levels than males (446 vs. 429 μmol/L; *p* < 0.001). However, these differences are not clinically meaningful given the comparable proportions of measurements within target.

In adulthood, significant sex-related differences were observed across all age groups for both mean Phe concentrations and the proportion of measurements within target. The largest disparity occurred in the 31–40-year age group, where only 10% of male measurements were within target compared with 69% among females.

### 3.5. Blood Phenylalanine Control by PKU Severity

**Figure 3:**
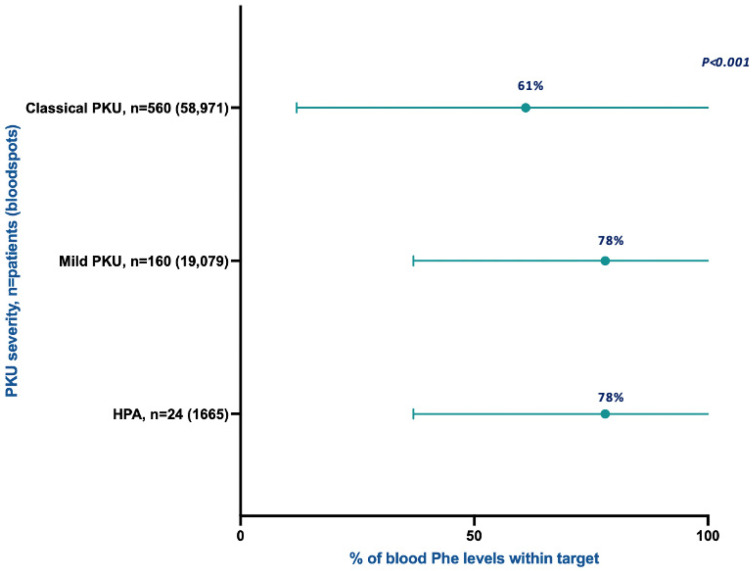
Mean percentage of blood Phe levels within therapeutic target range according to different PKU severities.

Mean blood Phe concentrations were 300 ± 110 μmol/L in patients with HPA, 309 ± 175 μmol/L in those with mPKU, and 386 ± 275 μmol/L in those with cPKU. No statistically significant differences were observed between HPA and mPKU in either mean Phe concentrations or the proportion of measurements within the therapeutic target range (78% vs. 78%; *p* = 0.141; Figure 3). In contrast, patients with cPKU had a significantly lower proportion of measurements within target (61%) compared with those with mPKU and HPA (78%) (*p* < 0.001; Figure 3).

### 3.6. Frequency of Blood Phe Monitoring

Table 5 presents mean blood Phe concentrations and the percentage of measurements within the therapeutic target range according to the monitoring frequencies recommended by the European PKU guidelines. Overall, mean blood Phe concentrations increased as monitoring frequency decreased, and the proportion of measurements within target declined with less frequent sampling.

Mean blood Phe concentrations were lowest among patients who submitted weekly blood spot samples (254 ± 175 μmol/L). Mean Phe concentrations rose progressively with decreasing monitoring frequency, reaching a maximum of 624 ± 349 μmol/L among individuals submitting samples less than once per month.

The proportion of measurements within the therapeutic target range followed the same pattern: 82% with weekly blood Phe sampling, decreasing to 70% with fortnightly sampling, 61% with monthly sampling, and 44% when samples were submitted less than once per month (*p* < 0.001).

### 3.7. Timing of Blood Phenylalanine Measurement

In 328 patients (38%), the time of blood spot collection was available. Samples were categorised as either morning (*n* = 5741) or afternoon (*n* = 5291). Mean blood Phe concentrations were higher in samples collected in the morning (411 ± 238 μmol/L) compared with those collected in the afternoon (347 ± 233 μmol/L).

**Figure 4:**
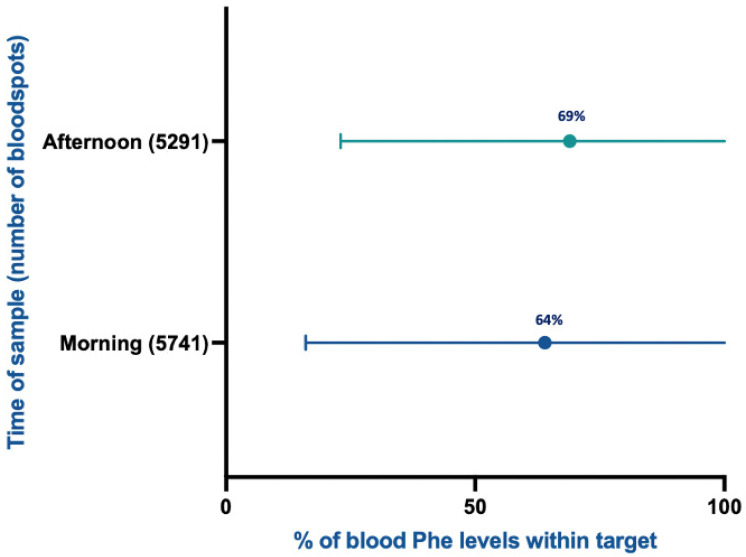
Percentage of blood phenylalanine levels within target range according to time of sampling: morning vs. afternoon.

Figure 4 presents the percentage of blood Phe concentrations within target performed in the morning compared with afternoon. Sixty-nine per cent of afternoon samples were within therapeutic target levels compared with 64% of morning samples.

### 3.8. Clinic Resources

All participating clinics were specialist PKU services. Both children and adults with PKU were represented in all UK regions. Multidisciplinary teams typically consist of physicians, dietitians, nurses and dietetic assistants. At the time of study, only one paediatric centre had a clinical psychologist as part of the multidisciplinary team.

All participating treatment centres had broadly comparable resources available during the study period (2012–2018). Special low-protein foods and protein substitutes were accessible throughout the UK via prescription, with costs reimbursed by the National Health Service (NHS) for children. For adults, prescription systems varied by country. In England, adults with PKU were required to pay the standard prescription fee per item unless they purchased a 12-month pre-payment certificate, which offered the most cost-effective option. Exemptions from prescription charges applied to individuals aged over 60 years, pregnant women and those on low incomes. In contrast, prescriptions were provided free of charge to all patients in Scotland, Wales and Northern Ireland.

## 4. Discussion

This retrospective study included data from 871 patients across the UK: 358 adults from six adult PKU centres and 513 children from nine paediatric centres. Overall, 61% of all patients cared for by these centres participated, with centre-level participation ranging from 20% to 100%. The proportion of blood Phe measurements within the therapeutic ranges defined by the PKU European guidelines varied widely, from 56% to 83%, and most centres reported fewer than 70% of results within the recommended target range. These findings are comparable to UK blood Phe control reported in 2002 [27], despite substantial advances in the range and type of protein substitutes and the increased availability of special low-protein foods.

As expected, blood Phe levels increased with age, with the proportion of measurements within target declining from 78% in children under 2 years to 36% in adults over 40 years. Across most centres, metabolic control deteriorated substantially during adolescence, despite the higher upper target threshold for this age group (600 µmol/L). This pattern was consistent across UK services and highlights a persistent vulnerability in metabolic management during the transition from childhood to adulthood. Notably, in our cohort the proportion of measurements within target fell below 70% as early as 6–12 years of age, substantially earlier than reported in European cohorts and consistent with findings from a Canadian cohort [34]. In contrast, European data [33] indicate that proportions below 70% typically occur only in adulthood, suggesting that UK children and adolescents may experience a steeper and earlier decline in metabolic control. Furthermore, while European cohorts [33] show an apparent improvement in metabolic control during adolescence (85% within target), largely attributable to the increase in the upper target threshold from 360 to 600 µmol/L, UK adolescents did not demonstrate a similar benefit. Instead, metabolic control continued to decline, with only 61% of measurements within target despite the same therapeutic range adjustment. This divergence raises important questions about structural, service-level, and behavioural factors that may differentially influence adherence and monitoring practices in the UK. This course is concerning given the extensive evidence linking elevated Phe exposure to neurocognitive and psychosocial outcomes across the lifespan [35,36]. The earlier and more pronounced deterioration observed in UK cohorts may therefore have important implications for long-term cognitive health, educational attainment, and quality of life.

All participating individuals were managed within specialist metabolic centres staffed by dedicated multidisciplinary teams, indicating that the suboptimal metabolic control observed in this study cannot be readily attributed to variation in service provision. However, with the exception of one centre, participating sites obtained consent to report blood Phe concentrations from only a subset of their clinic populations (mean participation was 61%). This introduces a substantial risk of selection bias. Individuals who consent to research participation are typically those who are more engaged with clinical care, more adherent to monitoring recommendations, and more responsive to communication from their metabolic teams. As a result, the dataset likely over-represents individuals with comparatively better metabolic control. Consequently, the actual level of metabolic control in the wider non-participating population is likely to be worse than reflected in our findings. This interpretation is supported by data from Altman et al. [37], who reported complete centre-level data from 2015–2017 in study centre M and demonstrated higher mean Phe concentrations in the full clinic cohort compared with the consented research subset. Their analysis also showed that less frequent blood sampling was strongly associated with higher Phe concentrations. These observations align with longstanding challenges in PKU epidemiology. National estimates of metabolic control almost invariably underestimate the true measure of poor control. Outside of research participation, many individuals with PKU experience persistent barriers to sustained engagement with their treatment plan. Those with infrequent monitoring, poor dietary adherence, or long-standing disengagement from clinical services are far less likely to respond to research invitations and so are unlikely to be represented in clinic-based research datasets. Among adults, non-participation may also reflect functional limitations, such as impaired executive function, organisational difficulties, or reduced capacity to manage administrative tasks, that are themselves consequences of chronically elevated Phe levels. These neurocognitive and psychosocial barriers may further reduce the possibility that adults with the poorest metabolic control engage in research activities. We also have no data on the number of adults lost to follow-up. Taken together, these factors suggest that the metabolic control that we report probably represents an upper-bound estimate of national performance.

It is also noteworthy that approximately half of the blood Phe samples in our dataset were collected in the afternoon, which may have resulted in lower measured blood Phe concentrations compared with fasting values. As most individuals were managed with dietary treatment alone, ingestion of protein substitutes throughout the day would be expected to suppress circulating Phe concentrations, thereby contributing to lower afternoon results [38,39]. This sampling pattern introduces a systematic bias that likely leads to an underestimation of true metabolic control challenges.

Elevated blood Phe concentrations are consistently associated with adverse neurocognitive outcomes across the lifespan in individuals with PKU [17,40]. Waisbren et al. [38] demonstrated a dose-dependent effect, with each 100 µmol/L increase in Phe corresponding to a 1.9–4.1-point reduction in IQ, and a recent systematic review confirmed that lower mean Phe concentrations are associated with higher IQ in both adults and children [39]. Evidence also shows that Phe concentrations >360 µmol/L during the first 12 years impair cognitive function [41] and are linked to reduced educational attainment [42]. These findings are directly relevant to our cohort, in which metabolic control fell below recommended targets from mid-childhood onwards and declined further during adolescence. Given that attention difficulties, including distractibility, forgetfulness, task interruptions, and organisational problems, have been documented in 8–17-year-olds with Phe >360 µmol/L [43], and that higher current Phe levels in this age group are associated with poorer executive functioning (slower reaction times, reduced working memory, impaired planning) [44], the early and sustained loss of control observed in our data is clinically concerning. The pattern suggests that a substantial proportion of UK children and adolescents may be exposed to Phe levels associated with measurable neurocognitive risk, reinforcing the importance of improving metabolic management during this critical developmental period.

Structural brain abnormalities are frequently reported in adults with early-treated PKU, with cerebral white matter changes particularly common and increasing in severity from the second decade of life onwards [45,46,47]. Cognitive consequences are well documented: in a cohort of early-treated adults, Tomm et al. [42] found executive function performance in the lower average range, with poorer outcomes associated with higher childhood Phe levels, current Phe concentrations, and Phe variability. Neurological complications, ataxia, tremor, clumsiness, epilepsy, and brisk reflexes, occur in an estimated 20–40% of early-treated adults and may improve with better metabolic control [48]. In the ECOPHEN study, neurological and psychiatric comorbidities were concentrated in adults with cPKU and markedly elevated Phe (mean 1156 µmol/L) [49], with depressive and anxiety symptoms also common [50]. These findings have direct relevance to our cohort, in which metabolic control declined from adolescence and remained suboptimal in adulthood. Given the strong associations between elevated Phe and structural, neurological, and psychiatric morbidity, the persistently high Phe concentrations observed in many UK adults likely place them at increased long-term risk. Our data therefore reinforce the clinical importance of targeting adult metabolic management to prevent neurocognitive and neurological complications.

International guidelines consistently endorse lifelong treatment for PKU, including those from Europe [7], the United States [51], Canada, and Australia [52]. Although all UK centres included in this study had adopted the European guideline targets at the time of data collection, some measurements in our dataset pre-dated guideline publication, and no patients had access to sapropterin, pegvaliase, or sepiapterin. Importantly, all but one of the specialist centres in our cohort did not have access to a neuropsychologist, despite the high prevalence of suboptimal metabolic control and the associated risk of neurocognitive and neuropsychiatric complications. This gap in multidisciplinary provision is concerning given our findings of declining metabolic control from adolescence into adulthood, and it highlights a critical unmet need within UK PKU services.

Several factors may have contributed to the suboptimal metabolic control observed in our cohort. Inconsistent communication about the consequences of elevated Phe remains a major barrier: in a UK adult survey, only 32% of respondents reported being informed about the risks of high Phe in adulthood [30]. High patient caseloads spanning multiple inherited metabolic disorders may further limit the time available for PKU-specific support, while involvement of multiple professionals can disrupt continuity of care and weaken therapeutic relationships, reducing opportunities for proactive follow-up and personalised intervention [53]. Infrequent physical and biochemical monitoring may also diminish engagement, particularly in adults. Broader societal and financial pressures, combined with limited practical support for families, further hinder adherence to a restrictive and burdensome diet. Older adults face additional challenges, as many were historically advised to discontinue dietary treatment during adolescence [54], complicating re-engagement with lifelong management in those now over 41 years of age. Finally, the UK has a higher proportion of individuals with cPKU compared with Southern Europe [6], which may contribute to poorer metabolic control at a population level.

Comparable to findings from European cohorts [33], our data show that more frequent blood Phe monitoring is associated with improved metabolic control. While this association may partly reflect that individuals with better adherence are also more likely to submit regular samples, behavioural mechanisms are also plausible. Regular monitoring may reinforce motivation, increase accountability, and heighten awareness of metabolic status, creating a “positive feedback loop” that supports sustained adherence. Conversely, infrequent sampling may contribute to an “out-of-sight, out-of-mind” pattern, reducing engagement and allowing blood Phe levels to drift upward without timely corrective action.

The current DBS-based home monitoring system has important limitations. Postal delays frequently prolong sample turnaround, restricting timely dietary adjustments and increasing variability in blood Phe concentrations [55]. These delays are particularly problematic in newly diagnosed infants and during intercurrent illness, when rapid biochemical feedback is essential. DBS quality is also inconsistent, issues such as inadequate saturation, layering, contamination, and environmental exposure can affect accuracy, and DBS Phe values are often lower than corresponding venous measurements [56]. Overall, DBS monitoring is burdensome and provides neither timely nor consistently reliable results. There is a clear need for rapid, accurate home Phe-testing tools, similar to established models in diabetes care [57,58], to enable more responsive dietary adjustments to improve metabolic control.

Although the UK has a structured transition pathway, transfer to adult services typically occurs between 16 and 18 years, an age already characterised by major educational, social, and geographic changes [59]. These disruptions coincide with a developmental stage in which adherence to dietary management and monitoring is particularly vulnerable. Evidence from other countries shows that loss to follow-up can occur even when paediatric and adult services are co-located [59], indicating that structural proximity alone does not ensure continuity of care. Our findings demonstrate a marked deterioration in metabolic control from mid-childhood, with a further decline during adolescence, suggesting that reduced monitoring and inconsistent attendance during transition may contribute to prolonged periods of elevated Phe. Given the strong association between regular monitoring and improved metabolic control, any reduction in specialist contact at this stage poses a significant risk. Ensuring seamless lifelong access to specialist PKU services is therefore essential to prevent avoidable deterioration in metabolic control and its long-term neurocognitive and psychosocial consequences [60].

Sex-related differences in metabolic control were evident in our cohort, contrasting with several European reports. Blood Phe concentrations differed significantly between males and females across most age groups, with males generally exhibiting poorer control, most strikingly in the 31–40 year group, where only 10% of male measurements were within target compared with 69% in females. These disparities may reflect differences in engagement with clinical services. Ilgaz et al. [30] reported that adult male patients often felt overlooked in adult care and received less detailed clinical reviews than female patients, suggesting a potential structural contributor to reduced monitoring and adherence. Our findings align with this pattern: males in multiple age groups demonstrated higher mean Phe levels, indicating greater vulnerability to disengagement and poorer metabolic stability. While optimising care for women, particularly during pregnancy, remains essential, these data highlight the need to address unmet needs among male patients to ensure equitable lifelong management. Additional factors may also contribute. Higher participation in high-intensity physical activity among males could increase catabolic stress if not matched by adequate energy and protein substitute intake, although this has not been systematically studied. Psychosocial and biological differences may further influence adherence and metabolic outcomes, highlighting the importance of tailored sex-sensitive approaches to PKU care.

This study has several limitations. Early-treated patients were defined as those who commenced dietary treatment by 3 months of age. All data were collected retrospectively from dietetic and medical records, which varied across centres and included both handwritten and electronic documentation. Not all centres had complete datasets, and recruitment numbers differed substantially between sites. The overall participation rate was 61% of eligible patients, raising the possibility of selection bias; individuals who are more adherent and engaged with clinical care may have been over-represented, potentially leading to an overestimation of metabolic control in the wider PKU population.

We were unable to determine if blood Phe samples were collected during periods of intercurrent illness or if monitoring frequency decreased during illness, both of which could influence Phe variability. No data were available on population demographics beyond age or on individual dietary adherence, limiting our ability to adjust for these factors. Information on the number of patients lost to follow-up was also unavailable, which may further underestimate the true extent of suboptimal blood Phe control in routine practice. Data collection occurred largely before the publication of the European PKU guidelines, and several centres may have been targeting higher blood Phe ranges, particularly in adulthood. At the time of data collection, no alternative pharmaceutical treatments were available in the UK outside of clinical trials, meaning dietary therapy was the only routinely accessible treatment option.

Differences between DBS and venous plasma Phe concentrations are well recognised, with dried blood spot values typically reported to be 10–25% lower than plasma measurements [61,62,63,64,65]. This discrepancy suggests that true Phe concentrations in our cohort may have been higher than those reported here. Analytical method bias, inter-laboratory variation even when identical methods are used, and the influence of DBS size and sample quality were not accounted for in this analysis. Furthermore, DBS samples were processed by eleven different laboratories, introducing additional variability into the reported Phe concentrations. Nearly half (47%) of samples with available timing data were collected in a non-fasting state. This represents an important limitation, as non-fasting samples may underestimate true Phe levels and could partly contribute to the variability observed in our dataset.

Mutation analysis data were unavailable for many participants. At the time of data collection, sapropterin was not routinely prescribed in the UK, and PAH genotyping was therefore not performed. As a result, we were unable to examine genotype–phenotype relationships or explore whether responsiveness to BH4 therapy might have influenced metabolic control.

Taken together, these limitations indicate that the findings should be interpreted with caution. Nonetheless, they provide valuable insights into UK metabolic control and service delivery during a period when dietary management was the only routinely available treatment option for PKU outside of clinical trials.

## 5. Conclusions

Overall metabolic control in individuals with PKU in the UK remains suboptimal and shows no meaningful improvement compared with data reported in 2002, despite the increased availability of dietary resources and updated clinical guidance. In this study, blood Phe control deteriorated progressively with age, and male patients consistently demonstrated higher Phe concentrations than females. These findings reinforce the importance of sustained engagement with dietary management and regular biochemical monitoring across the lifespan. The introduction of new adjunct therapies will be critical for improving metabolic control. Such therapies may offer opportunities to enhance neurocognitive outcomes and reduce, or potentially eliminate, the substantial burden associated with exclusive reliance on dietary treatment.

## Figures and Tables

**Table 1:** Centre characteristics: patients and number of blood spots for phenylalanine.

Centres (*N* of Patients)	*N* of Patients Under Follow-Up Meeting Inclusion Criteria	*N* of Eligible Patients Recruited	% of Eligible Patients Recruited	*N* of Blood Spots	Mean Overall *N* of Blood Spots Performed Per Patient/Year
**Paediatric centres**					
A	33	27	82	5177	27
C	81	76	94	8865	17
D	97	97	100	22,478	33
E	60	39	65	3367	13
G	50	34	68	2509	11
I	40	12	30	1208	14
J	129	82	64	12,459	22
K	144	37	26	6021	23
N	89	34	38	5729	25
Adult centres *					
B	200	104	52	3297	5
F	20	8	40	300	5
H	40	22	55	787	5
L	200	40	20	2829	10
M	300	129	40	3115	4
O	150	79	53	3520	6
**Paediatric and Adult centres**					
P	70	51	73	7801	22

Abbreviations: *N*, number. * Adult centres also include patients that were classified as children throughout data analysis. Patients < 18 y were classified as children.

**Table 2:** Blood phenylalanine control and number of patients participating in this study by age category.

Age Category	*N* of Patients	*N* of Blood Spots	Mean ± SD		
			Blood Phe (μmol/L)	% Blood Phe Within Target Range	*p*-Value
Children	513	62,479	303 ± 199	71 ± 46	<0.001
Adults	358	29,387	491 ± 308	59 ± 49	

Abbreviations: *N*, number; Phe, phenylalanine; SD, standard deviation.

**Table 3:** Mean blood Phe and percentage (%) of Phe levels within target range per age group.

Age Group (Years)	*N* of Patients	*N* of Blood Spots	Mean ± SD	
			Blood Phe (μmol/L)	% Blood Phe Within Target Range
<2	69	6786	272 ± 226	78 ± 41
2–5	97	17,184	285 ± 206	75 ± 43
6–12	219	42,819	317 ± 188	67 ± 47
13–18	128	13,429	437 ± 234	61 ± 49
19–30	164	5436	596 ± 351	57 ± 50
31–40	108	3679	559 ± 375	62 ± 49
≥41	86	2431	750 ± 362	36 ± 48

Abbreviations: *N*, number; Phe, phenylalanine; SD, standard deviation.

**Table 4:** Mean blood phenylalanine concentrations and percentages of blood phenylalanine concentrations within target per age group comparing males and females.

Age Group (Years)	Females				Males				*p* Value
	*N* of Patients	*N* of Blood Spots	Blood Phe (μmol/L)	% Blood Phe Within Target Range	*N* of Patients	*N* of Blood Spots	Blood Phe (μmol/L)	% Blood Phe Within Target Range	
<2	26	2513	278 ± 237	78 ± 42	43	4273	268 ± 219	78 ± 41	0.387
2–5	47	8168	283 ± 205	75 ± 43	50	9016	287 ± 206	75 ± 43	<0.001
6–12	108	20,927	315 ± 194	68 ± 47	111	21,892	319 ± 182	67 ± 47	0.212
13–18	61	6197	446 ± 246	61 ± 49	67	7232	429 ± 222	61 ± 49	<0.001
19–30	107	3897	584 ± 350	58 ± 49	57	1539	628 ± 351	54 ± 50	<0.001
31–40	67	3235	501 ± 339	69 ± 46	41	444	987 ± 344	10 ± 30	<0.001
≥41	62	1680	729 ± 375	40 ± 49	24	751	799 ± 327	26 ± 44	<0.001

Abbreviations: *N*, number; Phe, phenylalanine; SD, standard deviation.

**Table 5:** Mean blood Phe levels according to frequency of blood spot monitoring.

Monitoring Frequency	*N* of Patients	*N* of Blood Spots	Mean ± SD		*p* Value
			Blood Phe (μmol/L)	% Blood Phe Within Target Range	
Weekly	86	15,633	254 ± 175	82 ± 38	
Once every 2 weeks	231	45,339	319 ± 207	70 ± 46	<0.001
Once every 4 weeks	169	19,253	397 ± 231	61 ± 49	<0.001
>4 weeks	385	11,641	624 ± 349	44 ± 50	<0.001

Abbreviations: *N*, number; Phe, phenylalanine; SD, standard deviation.

## Data Availability

The original contributions presented in this study are included in the article. Further inquiries can be directed to the corresponding author.
